# Factors associated with internal medicine physician job attitudes in the Veterans Health Administration

**DOI:** 10.1186/s12913-018-3015-z

**Published:** 2018-04-05

**Authors:** David C. Mohr, Jennifer L. Eaton, Mark Meterko, Kelly L. Stolzmann, Joseph D. Restuccia

**Affiliations:** 1 Center for Healthcare Organization and Implementation Research (CHOIR), Boston VA Healthcare System, 150 South Huntington Ave, 152M, Boston, MA 02130 USA; 2 VA Office of Reporting, Analytics, Performance, Improvement and Deployment (RAPID –10EA), Field-based at the Edith Nourse Rogers Memorial Veterans Hospital, Bedford, MA USA; 3 Boston University School of Public Health, Boston, MA USA; 4 Department of Veterans Affairs, Office of Patient Care Services, Occupational Health Services, Washington, DC USA; 5 Boston University Questrom School of Business, Boston, MA USA

**Keywords:** Physician satisfaction, Physician burnout, Research involvement, Veterans affairs

## Abstract

**Background:**

US healthcare organizations increasingly use physician satisfaction and attitudes as a key performance indicator. Further, many health care organizations also have an academically oriented mission. Physician involvement in research and teaching may lead to more positive workplace attitudes, with subsequent decreases in turnover and beneficial impact on patient care. This article aimed to understand the influence of time spent on academic activities and perceived quality of care in relation to job attitudes among internal medicine physicians in the Veterans Health Administration (VHA).

**Methods:**

A cross-sectional survey was conducted with inpatient attending physicians from 36 Veterans Affairs Medical Centers. Participants were surveyed regarding demographics, practice settings, workplace staffing, perceived quality of care, and job attitudes. Job attitudes consisted of three measures: overall job satisfaction, intent to leave the organization, and burnout. Analysis used a two-level hierarchical model to account for the nesting of physicians within medical centers. The regression models included organizational-level characteristics: inpatient bed size, urban or rural location, hospital teaching affiliation, and performance-based compensation.

**Results:**

A total of 373 physicians provided useable survey responses. The majority (72%) of respondents reported some level of teaching involvement. Almost half (46%) of the sample reported some level of research involvement. Degree of research involvement was a significant predictor of favorable ratings on physician job satisfaction and intent to leave. Teaching involvement did not have a significant impact on outcomes. Perceived quality of care was the strongest predictor of physician job satisfaction and intent to leave. Perceived levels of adequate physician staffing was a significant contributor to all three job attitude measures.

**Conclusions:**

Expanding opportunities for physician involvement with research may lead to more positive work experiences, which could potentially reduce turnover and improve system performance.

**Electronic supplementary material:**

The online version of this article (10.1186/s12913-018-3015-z) contains supplementary material, which is available to authorized users.

## Background

Physician job satisfaction has recently emerged as a key performance indicator for US healthcare organizations. Job attitudes among physicians are linked to both healthcare system performance and workforce sustainability [1]. With increasing uptake of health insurance under the Affordable Care Act, it is predicted that demand will outstrip supply across the US healthcare system [2–4]. Physician job satisfaction, turnover and burnout are increasingly important indicators of looming supply side problems, particularly for healthcare organizations which often have multiple missions, including clinical care, research and teaching. At the organizational level, benefits of academic activity can include promotion of quality improvement and translation of research into practice [5]. Research-oriented medical centers exhibited similar lengths of stay [6] and lower risk-adjusted mortality rates [7] in comparison to non-research medical centers. Further, physician engagement in research may lead to improved quality of care, improved clinical efficacy, and effectiveness [8]. Despite the potential benefits of research involvement, the number of physicians conducting research as part of their regular work activities appears to be declining [9]. Several studies have examined barriers to research involvement, which can include time constraints and workload [10, 11]. Institutional factors can also limit research mentoring and support for physician-scientists [12, 13].

Physician job attitudes are a longstanding strategic concern for hospitals and professional organizations [14]. Studies have found significant relationships between physician job attitudes and quality of patient care, physician errors, [15] turnover, [16] patient satisfaction, [17] and adherence to formal care guidelines [18]. Studies also have shown positive relationships between research involvement and job attitudes among physicians in academic practice [19]. Overall job satisfaction among academic faculty appears to be low, with several studies demonstrating high overall job dissatisfaction, attrition, and burnout [20]. Involvement with research and teaching may allow physicians greater work autonomy and increase collaboration opportunities, [21] which may lead to higher work engagement and job satisfaction. In one international study, most physicians perceived research involvement to be helpful in their profession and had a positive opinion of research activities [22]. Prior work among US physicians suggests that highly satisfied physicians may be more likely to spend significant time in teaching and research activities [23].

Job attitudes have been widely studied and models to understand can be classified into three groups: (1) dispositional models, which consider biological, personality and other worker characteristics; (2) cross-cultural models, which consider differences by countries or cultures; and (3) work or organization-specific models, which consider various aspects of the job and working conditions [24]. While several frameworks have been proposed and refined through research, popular models within the work specific framework include the job characteristics model [25] and job the demands-resources model [26]. The job characteristic model focuses on internal factors to the work itself, such as skill variety, autonomy, and feedback, while the job demands-resources model focuses on external factors to the work itself, such as amount of work, pressure, promotion, coworkers and supervisors. For this study, we selected variables that reflected a.) individual professional characteristics, such as tenure and specialization [27, 28]; b.) internal factors, such as task variety and perceived quality of care, [29, 30]; and c.) external factors, such as staffing levels, [31, 32], and financial incentives [33].

### Study purpose

The context of the study is the Veterans Health Administration (VHA). VHA has an academic mission. The VHA has active affiliation agreements with more than 80% of US medical schools, training more than 100,000 health professional students and residents each year [34]. Prior work found VHA physicians who were involved with research reported more favorable workplace environment perceptions [35] and that research opportunities were a major factor facilitating recruitment and retention [36]. Thus, we hypothesize that greater levels of research involvement will relate positively to job attitudes among VHA physicians. We extend prior research findings by considering the multi-level influence of individual- and organizational-factors on internal medicine physician job satisfaction, intention to leave, and burnout.

## Methods

### Participants and design

We used a cross-sectional design and included inpatient medicine physicians at 36 acute care VA medical centers (VAMCs). The subset of VAMCs was randomly selected from 124 hospitals with acute-care inpatient services, stratified by geographic location and size. The Inpatient Medicine Physician Survey consisted of 39 questions covering four major content areas: work activities, perceptions and evaluations of patient care, coordination practices, and demographics. Data were collected as part of a larger research study examining organizational factors affecting the quality and cost of VA inpatient medical care. Physicians were emailed an invitation to participate during August and September 2010 with a link to a confidential, web-based survey. The survey was administered online and included some previously published measures (see Additional file 1) [37, 38]. Physicians received up to four email reminders sent at approximately two-week intervals over a ten-week period. Contact information was obtained from internal sources, including medicine unit work schedules and email directories. A total of 458 physicians responded for a response rate of 57%; however, due to missing values on outcomes, our final sample included 373 respondents.

### Ethics and consent to participate

The study was approved by the VHA Boston Healthcare System Institutional Review Board. If respondents consented to participate in the survey, they were directed to a web- based survey and could decline to answer questions or discontinue the survey.

### Outcome measures

The outcome measures were physicians’ self-reported overall job satisfaction, intention to leave, and burnout. *Overall job satisfaction* was assessed using the item “Compared to what you think it should be, what is your current overall level of satisfaction with your job?”. Responses to this question ranged from 1 for “Not at all Satisfied” to 5 for “Very Satisfied.” We used a single-item measure shown to have comparable validity to multi-item and multi-scale measures of job satisfaction [39, 40]. The measure has also shown a positive relation with the technical quality of care [41]. The discrepancy-based approach we used combines two questions that focus on the “current” and “ideal” or “preferred” state into one question by asking respondents to consider what their current level of care perceptions compared to ideal and may be preferable to methods asking about overall levels of satisfaction, which may not account for ideal states.

*Intention to leave* was assessed with the item “If I were able, I would leave my job because I am dissatisfied.” Response options ranged from 1 for “Strongly disagree” to 5 for “Strongly agree.” We used a single item measure of *burnout* from the Physician Worklife Study, [42] which has been validated against other measures of burnout, specifically with emotional exhaustion [43, 44]. Respondents indicated their level of burnout on a five-point scale ranging from 0 for “I enjoy my work. I have no symptoms of burnout” to 4 for “I feel completely burned out and often wonder if I can go on. I am at the point where I may need some changes or may need to seek some sort of help.” We also examined a dichotomously coded measure of burnout, which coded for any indication of burnout (i.e., a response of 2, 3, or 4) [45].

### Respondent characteristics and workplace perceptions

To assess variation in work activities, respondents were asked: “In the **past year**, out of your **VA** professional time only, on average, what percent of your time did you spend on each of the following activities? The total should add up to 100%.” Respondents provided percentages of time dedicated to specific activities including: direct patient care, education, administration, teaching, and research. Physicians indicated whether they had a specialty or subspecialty board certification in internal medicine or family practice; coded with a value of “1” for *specialist.* Physicians indicated their time spent during the last year working for: a.) VA hospital; b.) academic affiliate; or c.) other site. We coded for *VA-only* employment if respondents indicated 100% of their time was at a VA hospital. Respondents also provided their *tenure* as the number of years spent on the inpatient medicine service at their current facility. Respondents indicated if they attended medical school outside of the United States (yes or no).

We assessed workplace staffing support using two measures. *Physician staffing* was assessed using the item “There is adequate physician staffing in inpatient medicine” and *nurse staffing* was assessed using the item “There are enough registered nurses to provide quality patient care in inpatient medicine.” Physicians provided their level of agreement using a 1 to 5 point Likert scale ranging from “Strongly Disagree” to “Strongly Agree.” *Perceived quality of care* was assessed using the item “How would you rate patient care at your hospital currently, compared with what you think it should be?” The response options ranged from 0 for “well below expectations” to 4 for “well above expectations.”

### Organization characteristics

We modeled organizational-level variables to control for their potential impact on outcomes. We modeled *inpatient bed size* as a continuous variable; *urban or rural* area as a dichotomous variable; and *teaching hospital affiliation* using Council of Teaching Hospitals (COTH) membership as a dichotomous variable. The Chief of Medicine (COM) at each facility also completed a survey on organizational factors. From their responses, we created a *performance-based compensation* variable to indicate if compensation was awarded a.) based on individual, team or combined individual and team performance or b.) not at all.

### Statistical analysis

We first examined descriptive statistics and assessed distribution properties of measures for normality. We created hierarchical linear models that included the physician-level variables as level-one predictors and organization-level characteristics as level-two predictors to account for physicians clustered within medical centers [46]. We regressed job satisfaction, burnout, and intent to leave on the full set of individual and organizational factors. Standardized and unstandardized coefficients with standard errors are reported. SAS version 9.2 (Cary, NC) was used for data analysis.

## Results

Respondent demographics and organizational characteristics are presented in Table 1. The average time spent on direct patient care was 63%. The average tenure on the unit was approximately 10 years. A majority of the respondents (74%) indicated they worked only at the VA and less than a quarter of respondents (23%) had attended a foreign medical school. Mean values for the three job attitude outcomes were 2.96 (SD = 1.00) for overall job satisfaction; 2.14 (SD = 1.06) for intention to leave and 0.98 (SD = .84) for burnout.

**Table 1:** Descriptive characteristics of individual respondents (*n* = 373) and organizational characteristics (*n* = 36)

Measure	Mean or N	SD or %
Individual level		
Percent of VA professional time spent		
Direct patient care	63.17	27.28
Administration	12.74	16.68
Attending educational programs	3.41	5.30
Education	7.38	9.65
Research	13.29	22.53
Specialist board certification/eligible	346	93%
Work only at VA	276	74%
Tenure at current facility	9.87	9.10
Foreign medical school	86	23.1%
Adequate physician staffing	3.37	1.08
Adequate registered nurses	2.78	1.10
Perceived quality of care	1.88	.84
Outcomes		
Overall job satisfaction	2.96	1.00
Intent to leave	2.14	1.06
Burnout	.98	.84
Organization-level		
Urban location	29	80.6%
Teaching affiliated hospital	25	69.4%
Performance-based compensation	26	72.2%
Inpatient beds	65.58	35.57

In the regression models (Table 2), employees who worked only at VA reported significantly higher burnout rates (β = 1.77). Respondents who reported more favorable scores for physician staffing reported significantly higher levels of overall job satisfaction (β = 4.43) and significantly lower ratings on intent to leave (β = − 4.48) and burnout (β = − 4.31). A similar finding was observed for the dichotomized measure of burnout. A positive association was observed for physicians’ perceived quality of care with overall job satisfaction (β = 6.35) and a negative association was observed between perceived quality of care and intention to leave (β = − 6.50). Physicians who spent more time on research activities reported higher overall job satisfaction (β = 2.08) and lower intention to leave (β = − 1.93).

**Table 2:** Hierarchical linear model regression for factors associated with percentage of time spent on research and job attitudes

	Job satisfaction		Intention to leave				Burnout		
	β	Unstd B	Std Err	β	Unstd B	Std Err	β	Unstd B	Std Err
Intercept	2.95^**^	.59	.30	2.14^**^	4.24	.32	.97^**^	2.16	.26
Research time	2.08^*^	.01	.00	−1.93^*^	−.01	.00	−1.55	.00	.00
Teaching time	1.48	.01	.00	−1.49	−.01	.01	−.26	.00	.00
Administrative time	.24	.00	.00	−.32	.00	.00	.61	.00	.00
Training time	−.05	.00	.01	.28	.00	.01	−.40	.00	.01
Board certified	.79	.18	.20	.13	.03	.21	.58	.13	.18
Work only at VA	−.85	−.10	.11	1.29	.15	.02	1.77^*^	.21	.10
Tenure at current facility	.06	.00	.00	.10	.00	.01	−.69	.00	.00
Foreign medical school	.24	.03	.11	.20	.02	.12	−.64	−.08	.10
Adequate physician staffing	4.43^**^	.21	.05	−4.48^**^	−.22	.05	−4.31^**^	−.21	.05
Adequate nurse staffing	−.83	−.04	.05	.33	.02	.05	−1.54	−.07	.04
Perceived quality of care	6.35^**^	.40	.06	−6.50^**^	−.40	.06	−1.04	−.06	.05
Urban setting	1.77	.26	.15	−1.00	−.15	.16	−1.33	−.19	.13
Teaching hospital	.97	.15	.06	−1.79	−.27	.18	.57	.08	.14
Performance compensation	.64	.07	.11	.46	.05	.12	−.63	−.06	.09
Inpatient bed size (per 100)	3.05^**^	.05	.02	−2.87^*^	−.05	.02	−1.61	−.02	.02

^*^ *p* < .05; ^**^ *p* < .01

Regarding organizational characteristics, inpatient bed size was significantly associated with the outcomes, showing a positive association with overall job satisfaction (β = 3.05) and a negative association with intention to leave (β = − 2.87) and burnout (β = − 1.61, ns). Performance compensation systems, urban setting, or teaching hospital affiliation were non-significant in relation to outcomes.

## Discussion

The study found physicians’ ratings on perceived quality of care and adequacy of physician staffing were the strongest predictors of overall job satisfaction and intent to leave. Among the job tasks that physicians spent their time on, time spent on research was associated with increased job satisfaction and decreased intent to leave.

Physicians reported job attitudes that were more favorable when they perceived greater levels of physician staffing on the unit, but the perception of having sufficient registered nurse staffing was non-significant with attitudes. While a great deal of literature has focused on nurse staffing and hours per patient day as they relate to patient outcomes, less attention has been spent on physician staffing in relation to patient outcomes. A focus on physician staffing ratios may represent a new area for further research for its influence on provider perceptions and patient care.

Providers’ quality of care expectations were strongly associated with job satisfaction and intent to leave. Hospitals where the overall care is not high quality may be at a disadvantage in recruiting and retaining physicians, especially as hospital ratings are publically available and likely known to physicians applying for positions at a health care organization. Negative publicity may affect not just a hospital’s public reputation, but its ability to recruit and retain clinical staff as well. Further research into how recruitment is affected by quality of care may be worthwhile.

We found the extent of research involvement was associated with favorable scores on outcomes. The finding may support the practice of allowing protected time for physicians to pursue research activities. VA has a long history of providing opportunities for research involvement and education activities in basic and applied care areas [47–49]. A major issue for policy makers is whether allowing research time for physicians, as well as other clinical staff, can provide benefits without taking away from patient care activities. VA formally identifies research as one of its four organizational missions, but research may not be seen as contributing to the mission of other health care systems. In some cases, research and academic orientation could be in conflict with strategic goals and objectives, such as increasing physician productivity. Whether or not the tradeoff between time spent on research and time spent on patient care is a zero-sum game is unclear and may not be a simple matter. For example, research activities may result from participation in quality improvement activities designed to improve patient care.

Hospitals are moving toward hiring more physicians as a potential mechanism for improving efficiency [50]. As this trend continues and hospitals have more direct control over their physician work activities, hospital management will have an opportunity to develop and shape policies that support research involvement. Management may want to include protected research time as part of their recruitment and retention strategy. Further exploring the ideal amount of research desired by clinicians would be informative. Some clinicians may prefer doing only clinical care and would not be interested in research, while others may prefer a blend of research and clinical care.

### Limitations

We note several limitations of the study. We used a cross-sectional design that limits causal inferences. Our study used single-item measures to represent several key constructs. While single-item measures have advantages over multiple-item scales, such as shorter survey length and comparable validity estimates, the present results could differ if established multi-item scales to measure psychological constructs and been used. Additionally, an opinion-based attitude survey is limited to a strict set of responses. Qualitative interviews with respondents about workplace factors and their attitudes about specific aspects of their work would yield an enriched dataset on which to draw conclusions. Additionally, the study was conducted within the VA healthcare system, so caution in generalizing results are warranted due to differences in billing, the predominance of salaried physicians, use of service agreements often requiring that tests be conducted before referral, and financial incentives for coding, among other factors.

Future research on issues relating more specifically to research activities and career trajectories appears warranted [51, 52]. For example, studies could explore whether physicians involved with research are more likely to provide better patient care, advance professionally in the organization, use resources more efficiently, or less likely to leave.

Interviews or surveys of the Chief of Medicine and other leaders on existence of policies and climate for research are also worth studying further. Detailed provider-level analysis over multiple periods examining the association between research involvement and outcomes, such as patient satisfaction and technical quality of care, would provide information to help answer questions regarding the trade-off between research and clinical time.

## Conclusions

Physician job attitudes are an increasingly important organizational performance indicator, with clear relationships to clinical quality, safety and provider supply. Recruitment and retention of healthcare professional is a critical driver of access and quality problems in VHA and many other organizations. As a federally-sponsored, national healthcare system VHA is comparable to international models and an important benchmark setting for US healthcare managers to understand. Research involvement, perceived adequate physician staffing and quality of care appear to have a favorable influence on multiple measures of job attitudes.

## Additional file

Additional file 1: Inpatient Medicine Staff Physician Survey. A copy of the survey instrument administered to participants is included. (DOC 187 kb)

## Abbreviations

COM: Chief of medicine
COTH: Council of teaching hospitals
VAMC: Veterans administration medical center
VHA: Veterans health administration
