# Supplementary material for: Factors associated with internal medicine physician job attitudes in the Veterans Health Administration
Source: BMC Health Serv Res. 2018 Apr 5;18:244. doi: 10.1186/s12913-018-3015-z (PMC5885351; doi:10.1186/s12913-018-3015-z)
Supplement: Supplementary file 1 — Inpatient Medicine Staff Physician Survey. A copy of the survey instrument administered to participants is included. (DOC 187 kb) [file 12913_2018_3015_MOESM1_ESM.doc]

**Inpatient Medicine Staff Physician Survey**

**Section 1. Distribution of Activities.**  These first questions ask about the amount of time you have spent in the past and currently spend doing various activities.

1. Are you currently a staff (attending) physician?

=1 Yes, I am currently a staff physician

=0 No, I am not currently a staff physician

1. How many years have you been a **staff (attending) physician**?
   1. On the inpatient medicine service at your current VA facility (years):____
   2. At any VA facility in any service (years):___
   3. At any VA or non-VA facility (years):_____
2. In the **past year**, out of **all** of your professional activities -- clinical, research, administrative, teaching, consulting, moonlighting –what percent of your time did you spend working at (total should equal: 100%):
   1. A VA Hospital:_________
   2. A VA Academic Affiliate: ________
   3. Other Site %: _______
3. In the **past year,** out of your **VA** professional time only, on average, what percent of your time did you spend on each of the following activities? (If you have been a staff (attending) physician for less than a year, please answer based on the period since you became a staff (attending) physician. The total should add up to 100%).

a. Direct Patient Care: **Inpatient^1^: medicine service**

b. Direct Patient Care: **Inpatient^1^: any other service** (e.g. medicine consult, subspecialty, etc.)

c. Direct Patient Care: **Urgent care/emergency department care^2^**

d. Direct Patient Care: **Ambulatory**^3^

e. Attending educational programs aimed at maintaining/improving clinical skills/delivery of patient care

f. Administration: Performing reporting requirements

g. Administration: Managing a program within a department, service or hospital; doing service, hospital-wide or national committee work

h. Education^4^

i. Research^5^

Total: 100%

^1^ preparing for and providing clinical care and follow up for patients; supervising house staff residents/medical students who provide clinical care

^2^ preparing for and providing clinical care and follow up for patients; supervising house staff residents/medical students who provide clinical care

^3^ preparing for and providing clinical care and follow up for patients; supervising house staff residents/medical students who provide clinical care

^4^ time spent providing formal, didactic education; working on medical school committees; or managing a resident, fellow or other student teaching program

^5^ working on IRB approved research projects; serving on a research committee, study section or grant approving board; writing grants/publications; presenting research findings at research meetings

1. In a typical 7 day week that you attended on the VA inpatient medicine service in the past year, on average what was the **total** number of patients you admitted over that entire period?
   1. Less than 5 (=1)
   2. 5-14 (=2)
   3. 15-24 (=3)
   4. 25-34 (=4)
   5. 35 or more (=5)
2. In a typical 7 day week that you attended on the VA inpatient medicine service in the past year, on average what was your **daily** census?
   1. Less than 5 (=1)
   2. 5-9 (=2)
   3. 10-14 (=3)
   4. 15-19 (=4)
   5. 20-24 (=5)
   6. 25 or more (=6)
3. In the past year, how often have both inpatient medicine nurses and inpatient medicine physicians worked together on the same quality improvement committees and/or teams?
   1. Never (=0)
   2. Rarely (=1)
   3. Sometimes (=2)
   4. Usually (=3)
   5. Always (=4)
   6. Don’t know (= -1)
4. Please check the response that best describes the status of each of the following activities at your VA inpatient medicine service during the past year.

| Activity | Status of this activity on the VA **inpatient medicine service** | | | | |
| --- | --- | --- | --- | --- | --- |
|  | Not Used  =0 | Used, but I don’t use or participate  =1 | I used or participated  =2 | I used or participated, and I helped develop and/or promote it  =3 | Don’t Know / Not Sure  =-1 |
| 1. Work process redesign or re-engineering (e.g. Six Sigma or Lean or Rapid Process Improvement Workshops (RPIW)) |  |  |  |  |  |
| 1. Activities to improve workforce recruitment, retention, and development |  |  |  |  |  |
| 1. Benchmarking **within** the hospital |  |  |  |  |  |
| 1. Benchmarking **with other** hospitals |  |  |  |  |  |
| 1. Learning best practices **from other** industries |  |  |  |  |  |
| 1. Patient flow improvement strategies |  |  |  |  |  |
| 1. Patient advisory groups |  |  |  |  |  |
| 1. Shared clinical governance by nurses and physicians |  |  |  |  |  |
| 1. Chronic disease registries |  |  |  |  |  |
| 1. Planned care for chronic illness (Wagner’s chronic disease model) |  |  |  |  |  |
| 1. Evidence-based practice guidelines/clinical pathways |  |  |  |  |  |
| 1. Disease-or condition-specific improvement projects |  |  |  |  |  |
| 1. Clinical (improvement) collaboratives |  |  |  |  |  |
| 1. Case manager, social worker or other clinical staff to coordinate or manage patient care |  |  |  |  |  |
| 1. Pharmacists placed in patient care units |  |  |  |  |  |
| 1. Going on multidisciplinary rounds |  |  |  |  |  |
| 1. Medication reconciliation |  |  |  |  |  |
| 1. Rapid response teams |  |  |  |  |  |
| 1. Taking actions to prevent adverse drug events |  |  |  |  |  |
| 1. Taking actions to prevent central line infections |  |  |  |  |  |
| 1. Taking actions to prevent decubitus ulcers |  |  |  |  |  |
| 1. Taking actions to prevent falls |  |  |  |  |  |
| 1. Taking actions to prevent surgical site infections |  |  |  |  |  |
| 1. Taking actions to prevent ventilator-associated pneumonia |  |  |  |  |  |

**Section 2. Perceptions and Evaluations.**  The next questions ask about various aspects of patient care on the inpatient medicine service at your facility, and your perceptions regarding handoffs and coordination of care, the nature and extent of quality improvement activities and your overall impressions of hospital performance. Some of the questions ask for factual information; others ask for your evaluations.

1. During the past year, in a **typical 7 day week** that you attended, please list each location, type of location, and the approximate percentage of your patients admitted to that location.

| Name | Type of location (have drop down menu of medicine; surgery; med-surg; ICU and other | % of my patients admitted to that location |
| --- | --- | --- |
| Example: Ward 2 | Medicine | 30% |
|  |  |  |

1. In the **last month** you spent as a staff (attending) physician on the inpatient medicine service, how would you rate the following aspects of the coordination of patient care **during the inpatient stay**?

|  | Poor  =1 | Fair  =2 | Good  =3 | Very Good  =4 | Excellent  =5 | Not Applicable=0 | Don’t Know/ No Opinion=-1 |
| --- | --- | --- | --- | --- | --- | --- | --- |
| 1. Between physicians at admission |  |  |  |  |  |  |  |
| 1. Between nurses at admission |  |  |  |  |  |  |  |
| 1. Between physicians and nurses during the inpatient stay |  |  |  |  |  |  |  |
| 1. Between nurses during the inpatient stay |  |  |  |  |  |  |  |
| 1. Between medicine physicians during the inpatient stay |  |  |  |  |  |  |  |
| 1. Between medicine physicians and other inpatient consulting services (e.g., surgery, psychiatry) |  |  |  |  |  |  |  |
| 1. At transfer to and from the MICU |  |  |  |  |  |  |  |
| 1. With pharmacy services |  |  |  |  |  |  |  |
| 1. With social work services |  |  |  |  |  |  |  |
| 1. Inpatient coordination Overall |  |  |  |  |  |  |  |

1. In the **last month** you spent as a staff (attending) physician on the inpatient medicine service, how would you rate the following aspects of the coordination of patient care **related to the discharge process**?

|  | Poor  =1 | Fair  =2 | Good  =3 | Very Good  =4 | Excellent  =5 | Not Applicable=0 | Don’t Know/ No Opinion=-1 |
| --- | --- | --- | --- | --- | --- | --- | --- |
| a. Between physicians and patient/family |  |  |  |  |  |  |  |
| b. Between other staff and patient/family |  |  |  |  |  |  |  |
| c. Between inpatient medicine and primary care |  |  |  |  |  |  |  |
| d. Between inpatient medicine and specialty care |  |  |  |  |  |  |  |
| e. Between inpatient medicine and skilled nursing or rehab care (e.g. VA community living centers, skilled or intermediate facilities) |  |  |  |  |  |  |  |
| f. With pharmacy services |  |  |  |  |  |  |  |
| g. With social work services |  |  |  |  |  |  |  |
| h. Discharge coordination overall |  |  |  |  |  |  |  |

If no hospitalists are listed for the facility then in Q12 options a, will not appear and for b, the option will read ‘attendings’ rather than ‘non-hospitalist attendings’**

1. Please rate your agreement with the following statements. Hospitalists are defined as physicians whose primary professional focus is the general medical care of hospitalized patients. Their activities include patient care, teaching, research, and leadership related to hospital medicine.

|  | Strongly Disagree  =1 | Disagree=2 | Neither Agree nor Disagree  =3 | Agree  =4 | Strongly Agree  =5 | Don’t Know/No opinion  =-1 |
| --- | --- | --- | --- | --- | --- | --- |
| 1. Hospitalist attendings are available to nursing staff on the inpatient medicine service when nurses need them. |  |  |  |  |  |  |
| 1. Non-hospitalist attendings are available to nursing staff on the inpatient medicine service when nurses need them. |  |  |  |  |  |  |
| 1. Nursing staff are available to physicians on the inpatient medicine service when physicians need them. |  |  |  |  |  |  |
| 1. Inpatient nurses and inpatient medicine physicians work well together on quality improvement committees/teams. |  |  |  |  |  |  |

1. All frontline care providers in inpatient medicine function well as an interdisciplinary team.
   1. Strongly disagree (=1)
   2. Disagree (=2)
   3. Neither agree nor disagree (=3)
   4. Agree (=4)
   5. Strongly Agree (=5)

Please rate your agreement with the following statements regarding organizational support:

|  | Strongly Disagree  =1 | Disagree  =2 | Neither Agree nor disagree  =3 | Agree  =4 | Strongly Agree  =5 |
| --- | --- | --- | --- | --- | --- |
| 1. There is adequate **physician staffing** in inpatient medicine |  |  |  |  |  |
| 1. There are enough **registered nurses** to provide quality patient care in inpatient medicine. |  |  |  |  |  |
| 1. Employees in my work group have the appropriate **supplies, materials and equipment** to perform their jobs well. |  |  |  |  |  |
| 1. My facility provides appropriate continuing **education/training** to do my job. |  |  |  |  |  |

1. How much agreement do you perceive between the goals of senior leadership at the facility (quadrad) level and those of the inpatient medicine service?

No or almost no agreement (=0)
A little agreement (=1)
Some agreement (=2)
A great deal of agreement (=3)
Complete or almost complete agreement (=4)

1. How would you rate patient care at your hospital **currently**, compared with **three years ago**?

🔾 Much Worse (=0)

🔾 Worse (=1)

🔾 About the Same (=2)

🔾 Better (=3)

🔾 Much Better (=4)

🔾 Not here 3 years ago (=-1)

1. How would you rate patient care at your hospital **currently**, compared with **what you think it should be**?
   1. Well Below Expectations (=0)
   2. Below Expectations (=1)
   3. Meets Expectations (=2)
   4. Above Expectations (=3)
   5. Well Above Expectations (=4)

Please rate your agreement with the following statements:

|  | Strongly Disagree  =1 | Disagree  =2 | Neither Agree nor disagree  =3 | Agree  =4 | Strongly Agree  =5 |
| --- | --- | --- | --- | --- | --- |
| 1. A clear sense of direction exists among the senior leadership (quadrad) at this facility. |  |  |  |  |  |
| 1. Goals of senior leadership at the facility (quadrad) level and the inpatient medicine service are aligned. |  |  |  |  |  |
| 1. This facility is committed to the highest quality patient care. |  |  |  |  |  |

1. Compared to what you think it should be, what is your current overall level of satisfaction with your job?
   1. Not at all satisfied (=0)
   2. Not very satisfied (=1)
   3. Neither satisfied nor dissatisfied (=2)
   4. Somewhat satisfied (=3)
   5. Very Satisfied (=4)
2. If I were able, I would leave my current job because I am dissatisfied
3. Strongly disagree (=1)
4. Disagree (=2)
5. Neither agree nor disagree (=3)
6. Agree (=4)
7. Strongly agree (=5)
8. Using your own definition of burnout, which of the following best describes you currently (check one):
   1. I enjoy my work. I have no symptoms of burnout (=0)
   2. Occasionally I am under stress, and I don’t always have as much energy as I once did, but I don’t feel burned out. (=1)
   3. I am definitely burned out and have one or more symptoms of burnout, such as physical and emotional exhaustion (=2)
   4. The symptoms of burnout that I’m experiencing won’t go away. I think about frustration at work a lot. (=3)
   5. I feel completely burned out and often wonder if I can go on. I am at the point where I may need some changes or may need to seek some sort of help. (=4)

**Section 4. Background and Demographics.** This final group of questions asks for some basic background information. Please be reminded that the information you provide will be treated as strictly confidential; only aggregate results, not individual responses, will ever be reported.

1. For each phase of your medical training, please indicate the year you completed your training.

|  | Year Completed |
| --- | --- |
| Medical School |  |
| Residency |  |
| Fellowship |  |

1. Did you attend medical school in the United States?
   1. Yes (=1)
   2. No (=0)

1. Hospitalists are defined as physicians whose primary professional focus is the general medical care of hospitalized patients. Their activities include patient care, teaching, research, and leadership related to hospital medicine. By this definition, would you consider yourself a hospitalist?
   1. Yes (=1)
   2. No (=0)
   3. Not sure (=-1)
2. (If Yes to Q30) How many years have you worked as a hospitalist? ___ years
3. *Please indicate your specialty/subspecialty and board certification status. If your specialty/subspecialty does not have board certification, check N/A.*

|  | ***Board Certification*** | | | |
| --- | --- | --- | --- | --- |
| ***Specialty/Subspecialty*** | ***Yes (=1)*** | ***No***  ***(=0)*** | ***Eligible***  ***(=2)*** | ***N/A***  ***(=-1)*** |
| ***Internal Medicine (IM)*** |  |  |  |  |
| *IM: Addiction Medicine* |  |  |  |  |
| *IM: Adolescent Medicine* |  |  |  |  |
| *IM: Allergy & Immunology* |  |  |  |  |
| *IM: Bariatric Medicine* |  |  |  |  |
| *IM: Cardiovascular Disease* |  |  |  |  |
| *IM: Clinical & Lab Immunology* |  |  |  |  |
| *IM: Clinical Cardiac Electrophysiology* |  |  |  |  |
| *IM: Critical Care Medicine* |  |  |  |  |
| *IM: Endocrinology, Diabetes & Metabolism* |  |  |  |  |
| *IM: Gastroenterology* |  |  |  |  |
| *IM: Geriatric Medicine* |  |  |  |  |
| *IM: Hematology* |  |  |  |  |
| *IM: Hematology & Oncology* |  |  |  |  |
| *IM: Hepatology* |  |  |  |  |
| *IM: Hospice & Palliative Medicine* |  |  |  |  |
| *IM: Infectious Disease* |  |  |  |  |
| *IM: Interventional Cardiology* |  |  |  |  |
| *IM: Magnetic Resonance Imaging* |  |  |  |  |
| *IM: Medical Oncology* |  |  |  |  |
| *IM: Nephrology* |  |  |  |  |
| *IM: Pulmonary Disease* |  |  |  |  |
| *IM: Rheumatology* |  |  |  |  |
| *IM: Sleep Medicine* |  |  |  |  |
| *IM: Sports Medicine* |  |  |  |  |
| *IM: Transplant Hepatology* |  |  |  |  |
| ***Family Medicine (FM)*** |  |  |  |  |
| *FM: Addiction Medicine* |  |  |  |  |
| *FM: Adolescent Medicine* |  |  |  |  |
| *FM: Adult Medicine* |  |  |  |  |
| *FM: Bariatric Medicine* |  |  |  |  |
| *FM: Hospice and Palliative Medicine* |  |  |  |  |
| *FM: Sleep Medicine* |  |  |  |  |
| *FM: Sports Medicine* |  |  |  |  |
| *Other category (for double boarded)* |  |  |  |  |

1. Do you have any comments regarding the provision of inpatient care in the VA? Please include them here (open ended).

*Thank you very much for taking the time to complete this survey!*

*Peter Kaboli, MD, and Joseph Restuccia, DrPH*
